# Effects of vortioxetine on hippocampal-related cognitive impairment induced in rats by androgen deprivation as a model of prostate cancer treatment

**DOI:** 10.1038/s41398-023-02600-5

**Published:** 2023-10-03

**Authors:** Alexandra M. Vaiana, Yidong Chen, Jonathan Gelfond, Teresa L. Johnson-Pais, Robin J. Leach, Chethan Ramamurthy, Ian M. Thompson, David A. Morilak

**Affiliations:** 1 Department of Pharmacology, University of Texas Health Science Center, San Antonio, TX 78229 USA; 2 Center for Biomedical Neuroscience, University of Texas Health Science Center, San Antonio, TX 78229 USA; 3 Mays Cancer Center, University of Texas Health Science Center, San Antonio, TX 78229 USA; 4 Greehey Children’s Cancer Research Institute, University of Texas Health Science Center, San Antonio, TX 78229 USA; 5 Department of Population Health Sciences, University of Texas Health Science Center, San Antonio, TX 78229 USA; 6 Department of Urology, University of Texas Health Science Center, San Antonio, TX 78229 USA; 7 Department of Cell Systems & Anatomy, University of Texas Health Science Center, San Antonio, TX 78229 USA; 8 Department of Medicine, University of Texas Health Science Center, San Antonio, TX 78229 USA; 9 South Texas Veterans Health Care System, San Antonio, TX 78229 USA

**Keywords:** Hippocampus, Diseases, Molecular neuroscience, Pharmacology

## Abstract

Advances in prostate cancer treatment have significantly improved survival, but quality of life for survivors remains an under-studied area of research. Androgen deprivation therapy (ADT) is a foundational treatment for advanced prostate cancer and is used as an adjuvant for prolonged periods in many high-risk, localized tumors. More than half of patients treated with ADT experience debilitating cognitive impairments in domains such as spatial learning and working memory. In this study, we investigated the effects of androgen deprivation on hippocampal-mediated cognition in rats. Vortioxetine, a multimodal antidepressant, has been shown to improve cognition in depressed patients. Thus, we also tested the potential efficacy of vortioxetine in restoring impaired cognition after ADT. We further investigated mechanisms that might contribute to these effects, measuring changes in the circuitry and gene expression within the dorsal hippocampus. ADT via surgical castration induced impairments in visuospatial cognition on the novel object location test and attenuated afferent-evoked local field potentials recorded in the CA1 region of the dorsal hippocampus. Chronic dietary administration of vortioxetine effectively reversed these deficits. Castration significantly altered gene expression in the hippocampus, whereas vortioxetine had little effect. Pathway analysis revealed that androgen depletion altered pathways related to synaptic plasticity. These results suggest that the hippocampus may be vulnerable to ADT, contributing to cognitive impairment in prostate cancer patients. Further, vortioxetine may be a candidate to improve cognition in patients who experience cognitive decline after androgen deprivation therapy for prostate cancer and may do so by restoring molecular and circuit-level plasticity-related mechanisms compromised by ADT.

## Introduction

Over 50% of patients treated with androgen deprivation therapy (ADT) for prostate cancer will experience significant cognitive decline [1, 2]. As of 2022, prostate cancer is the second most common type of cancer in American men, exceeded only by basal cell and squamous cell carcinoma [3]. Unfortunately in the last 10 years, the incidence of prostate cancer has nearly doubled [3]. Despite high prevalence rates, prostate cancer is relatively treatable, with a 98% 5-year survival rate when diagnosed across all stages [3]. With this increase in survival, there is a need to optimize the quality of life for prostate cancer survivors.

For advanced stages of prostate cancer as well as high-risk localized tumors, ADT is a mainstay treatment, most often using gonadotropin-releasing hormone (GnRH) agonists or antagonists, reducing testosterone to castration levels. Unfortunately, the debilitating cognitive side effects induced by ADT can negatively impact day-to-day activities and quality of life for many patients [1, 4]. The effects of ADT on cognition initially present in the first 6–12 months after beginning treatment, and increase in severity with duration of treatment [5]. Cognitive domains that are primarily affected include executive function, attention, and memory [1, 2]. Functional imaging studies in humans have revealed significant changes in brain activity after ADT. BOLD-fMRI revealed that the parietal-occipital region shows reduced activation during recall of spatial information [6]. Follow-up studies found these patients had impaired performance in working memory tasks [7]. Particularly, these findings suggest significant impairment of visuospatial memory, which implicates dysfunction of the hippocampus [8, 9]. Although evidence suggests that ADT can induce profound cognitive impairment, many of the studies include small sample sizes or are largely observational. Furthermore, the cognitive endpoints are often different between studies, making it difficult to identify cognitive domains impacted consistently after ADT. Even fewer studies have investigated mechanisms that underlie these effects. Such studies are necessary to identify potentially novel and effective therapeutic targets for the treatment of cognitive impairment after ADT.

Vortioxetine is approved as an antidepressant by the U.S. Food & Drug Administration. Vortioxetine is unique in that it improves cognition in depressed patients by comparison to other selective-serotonin reuptake inhibitors (SSRIs) such as duloxetine [10–12]. In a large, placebo-controlled randomized study of depressed patients, vortioxetine was shown to improve overall cognition and performance in several specific cognitive domains, including executive function, attention, and processing speed, as well as learning and memory [13]. These effects were independent of its antidepressant effects [12]. Previously, our lab investigated ADT-induced cognitive impairments associated with the medial prefrontal cortex in rats and found that androgen depletion by surgical castration induced significant deficits in set-shifting, a measure of cognitive flexibility [14]. We also showed that chronic treatment with vortioxetine improved cognitive flexibility and reversed the attenuated electrical responses evoked in the mPFC by stimulation of afferent input from the ventral hippocampus in castrated rats. An important observation was that castration specifically altered the response to ventral hippocampal input but did not attenuate responsivity to another afferent projection to the mPFC from the medial dorsal thalamus, suggesting that the hippocampus may also have been affected. As previous clinical studies have implicated dysfunction of the hippocampus after ADT, and rodent studies have shown that testosterone depletion can alter neuronal morphology in hippocampus [8, 9, 15–17], this brain area might be implicated in the deficits that manifest in ADT patients, but this remains to be investigated. Vortioxetine has been shown to increase excitatory transmission [18] and enhance maturation of spines and dendrites in the hippocampus [19]. Therefore, we hypothesized that vortioxetine may be efficacious in improving cognitive changes associated with hippocampal dysfunction after ADT by improving or reversing changes in activity-dependent neuroplasticity that is necessary for optimal cognitive function. Furthermore, synaptic plasticity can be influenced by changes in gene expression. As the androgen receptor is a transcription factor, castration may contribute to the detrimental effects on cognition by dysregulating gene expression in the hippocampus. Vortioxetine has been shown to enhance activity in the hippocampus [20]. Thus, we hypothesized that vortioxetine may reverse changes in plasticity-related processes in the hippocampus induced by ADT. As vortioxetine is already in use clinically, the results of this investigation may suggest vortioxetine as a promising candidate by which to improve quality of life for cancer survivors by mitigating cognitive impairment after ADT.

## Methods

### Animals

As prostate cancer only affects males, these studies used only male rats. Young adult male Sprague-Dawley rats (Envigo, USA) were ~60 days old and weighed 225–250 g upon arrival. Rats from a given cohort were randomly assigned to treatment groups. For ADT treatment, animals were surgically castrated or received sham surgery and were separated into single housing 7–10 days before initiating treatment with drug-infused diet on experimental day 0. For the sham surgery, animals were anesthetized with isoflurane, the surgical site was prepared as usual, and a small incision was made in the scrotum, which was then closed with surgical glue. After either surgery, animals received subcutaneous injections of 1 mg/kg meloxicam and 10 mg/kg enrofloxacin then returned to their home cage. All testing or tissue collection occurred on day 17 post diet initiation. Housing conditions were as follows: for the novel object location (NOL) test and microarray experiments, animals were housed on a 12/12 hr light cycle (lights on at 7:00 h); for electrophysiology experiments, animals were on a 14/10 hr light cycle (lights on at 7:00 h). All animals were provided access to water and food ad libitum. Experiments were conducted during the light phase, starting by 9:00 and completed by 17:00 h. Separate cohorts of animals were used for each experiment. At the end of the experiment, plasma testosterone levels were measured via ELISA (IBL Inc., Minneapolis, MN, Kit # IB79174, inter-assay coefficient of variability = 3.95%). Plasma testosterone levels in castrated rats were below the detection limit of the assay (0.066 ng/mL), compared to intact controls = 2.34 ng/mL. All procedures were approved by the University of Texas Health San Antonio Institutional Animal Care and Use Committee and complied with National Institutes of Health guidelines.

### Vortioxetine treatment

Vortioxetine was obtained from H. Lundbeck A/S. Drug-infused chow containing 0.6 g vortioxetine per kg chow and control chow (Purina #5001) were prepared by Research Diets, Inc. Animals were placed on either control or vortioxetine diet beginning 7–10 days after castration and received drug for 17 days total. Free-feeding animals consumed about 7 g of food per 100 g body weight, corresponding to a dose of 40 mg/kg/day for an average 300 g rat. There were no differences in body weight between groups (castrated or sham-control rats receiving vortioxetine or control chow) at the time of testing, indicating no differences in food intake. This method of chronic vortioxetine administration has been well established by our group and others [14, 19, 21], and produces stable drug plasma levels within the range of therapeutic efficacy, with 60–95% occupancy at relevant drug targets in the brain [21].

### Open field test

The open field test (OFT) was used to assess locomotor activity as a potential confound for object exploration. Secondarily, the OFT provides a measure of anxiety-like behavior, another potential confound. The open field test occurred on day 16 and also served as habituation for the NOL test. The testing apparatus was a white wooden arena (65 × 65 × 42 cm^3^), with the floor marked into 36 squares (6 × 6) and covered by plexiglass. One of the walls was marked with 10 horizontal blue stripes to provide spatial orientation. The test was performed under light conditions of 6 lux. Total time in the arena was 20 min. The first 5 min were scored manually for total number of line crosses and time in the center squares of the arena, as measures of locomotion and anxiety-like behavior, respectively. All tests were recorded by an overhead GoPro® camera, and videos were analyzed offline by a blinded experimenter.

### NOL test

The NOL test was adapted from Barker & Warburton [22]. Testing (day 17) occurred 24 h after habitation (day 16). Animals were placed in the same testing apparatus as the OFT. Sample and test objects consisted of two identical Lego® figures (9.5 × 5.0 × 9.5 cm). The NOL test consisted of two phases. For the sample phase, two objects were placed in adjacent corners 10 cm from the walls and directly across from the striped wall. Animals were allowed 3 min to interact with both objects. After 3 min, the animals were removed from the arena and placed back in their home cage for a 5-min delay. During this time, one of the objects (the novel location object) was moved to an adjacent corner (nearest the striped wall) for the test phase. After the delay, the animals were returned to the arena for 3 min to complete the test phase. Interaction time with each object was measured, which included touching, sniffing, or facing the object within 2 cm. All objects and the arena were cleaned with 70% ethanol then water after each stage and between each animal. Interactions times (T) were scored for the familiar (*f*) and novel location objects (*n*) and calculated as a discrimination ratio [DR = (Tn−Tf)/(Tn+Tf)], in which values ranged from −1.0 to 1.0. Scores near zero indicate equal interaction time, thus a failure to recognize the new position of the object (i.e., a deficit of visuospatial memory). Scores closer to 1.0 indicate greater preference for the object in the novel location, hence better visuospatial memory of the object locations before they were moved.

### Afferent-evoked local field potentials in the CA1 region of dorsal hippocampus

Procedures for recording afferent-evoked local field potentials were adapted from previous studies [14, 23, 24]. Responses were recorded in the CA1 region of dorsal hippocampus after stimulating the Schaeffer Collaterals. Recordings occurred on day 17, the same day as behavioral testing in the preceding experiment. Animals were anesthetized using chloral hydrate (400 mg/kg, i.p. supplemented 10% as needed through the duration of recording) then placed in a stereotaxic apparatus. Body temperature was maintained at 37 °C. A stainless steel bipolar twisted stimulating electrode was positioned in the Schaeffer Collaterals, angled 30° toward the midline (coordinates from bregma: AP −3.8, ML + 5.0, DV: 4.0–4.7 mm). A tungsten recording electrode was then placed vertically in the ipsilateral CA1 region of the dorsal hippocampus (AP −3.8, ML + 2.0, DV −3.0 mm). The signal was filtered with a low cutoff at 0.3 Hz, high cutoff at 1000 Hz, and sampling at 2000 Hz. Signal was then digitized using PowerLab (ADInstruments). The response of the CA1 region was recorded after a 10-min equilibration period. Stimulus pulses, delivered in the Schaeffer Collaterals, were increased in 100 μA increments (100–800 μA, 0.1 Hz, 260 μsec pulse width). Magnitude of evoked responses were measured from the first negative peak, occurring at ~5–8 msec latency, to the first positive peak, at 12–15 msec. Data are presented as a current-response curve, using the average of 30 responses recorded at each stimulation intensity. Electrode placement was confirmed histologically, and cases in which electrodes were located outside of the targeted regions were excluded from the study.

### Whole genome microarray assessment of gene expression in the hippocampus

A separate cohort of animals treated identically to those described above but with no behavioral testing were sacrificed on day 17. The dorsal hippocampus was bluntly dissected on ice, and flash frozen in 2-methylbutane on dry ice. Samples were stored at −80 °C until use. Hemispheres were counterbalanced across experimental groups. Qiazol lysis buffer (Qiagen, Hilden, Germany) and Direct-zol miniprep RNA isolation kit (Zymo Research, Irvine, CA) were used to isolate RNA from the tissue. Samples with an RNA integrity number >7 were used for the assay, as determined using an Agilent Bioanalyzer (Agilent, Santa Clara, CA). mRNA expression patterns were evaluated using a whole genome microarray (Agilent G3 Rat GE 8 × 60 K v2 Microarray Kit, Agilent, Santa Clara, CA), as previously described [14]. Briefly, 100–200 ng of total RNA were used to create cyanine 3-labeled cRNA probes (Low Input Quick Amp Labeling kit, Agilent) that were hybridized to microarrays according to the manufacturer’s protocols (Agilent). For statistical analysis, differentially expressed genes (DEGs) were identified to test for main effects of castration and vortioxetine using the LIMMA R package (v3.46.0) [25], and utilizing Agilent Rat Microarray v2 gene annotation from NCBI/GEO (Platform GPL22145). DEGs were selected if they met criteria of adjusted *p* value < 0.01 and fold change (FC) > 2 or <−2. Gene Set Enrichment Analysis (GSEA; UC San Diego, San Diego, CA and the Broad Institute, Massachusetts Institute of Technology, Cambridge, MA) was performed by first selecting the top 60% most highly expressed genes (27,440 probes out of a total 45,738) and ranking them based on fold change (for a given comparison) to identify hallmark and KEGG pathways of interest [26, 27]. Significance was determined in GSEA by *q* < 0.05. Primary gene expression data files have been deposited in the Gene Expression Omnibus (GEO) database, accession number GSE236207.

### Statistical analyses

On the day of an experiment, the investigator was blind to treatment condition. Behavioral data were analyzed by 2-way ANOVA to determine main effects and interactions of ADT and vortioxetine (Castration x Drug) using GraphPad Prism 9 (San Diego, CA). Holm-Sidak test was used to detect differences between experimental conditions. In electrophysiology experiments, stimulus-response curves were compared using an extra sum-of-squares F-test [28]. Group sizes for electrophysiology and behavioral testing were determined a priori by power analysis, with power = 0.80, estimated mean difference = 35%, standard deviation = 25%. Significance was determined at *p* < 0.05 (two-tailed), unless otherwise noted (i.e., in the gene expression analyses above). For the NOL, animals were excluded from in the study if they failed to interact with one object entirely in the sample phase, or failed to interact with at least one object in the test phase. This resulted in the exclusion of 10 rats, and was not specific to any one treatment condition.

## Results

### Experiment 1: effects of androgen deprivation and vortioxetine on anxiety and locomotion

**Fig. 1:**
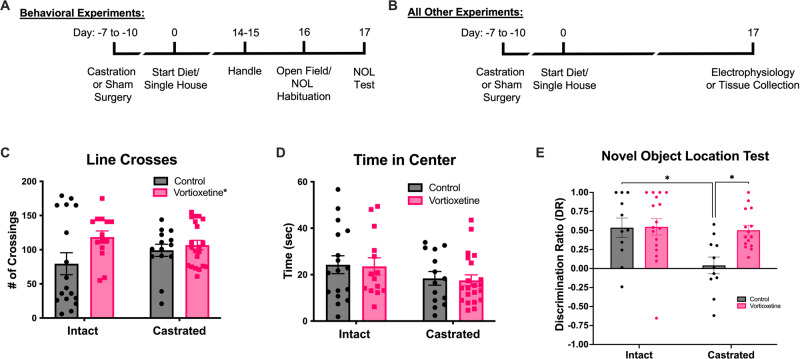
Behavioral effects of androgen deprivation and vortioxetine. Timeline of experimental procedure for **A** behavior, and **B** electrophysiology and tissue collection. Separate cohorts of rats were used for each experiment. Vortioxetine was administered in the diet for 17 days through the day of testing for all experimental measures. **C** Vortioxetine overall increased the total number of line crosses as a measure of locomotion on the open field test (*p < 0.05, *F*_1,62_ = 4.528), but pairwise comparisons between groups were not significant. **D** There were no differences in anxiety-like behavior, measured as time in the center of the arena. **E** On the NOL test, there was a significant decrease in the discrimination ratio (DR), indicating an impairment in visuospatial memory in surgically castrated rats compared to intact-control rats (*p < 0.05, *n* = 11/group). When castrated rats were administered vortioxetine chronically through the diet, the DR was restored to a level comparable to intact rats (*p* < 0.05 castrated/control vs castrated/vortioxetine, *n* = 10–11/group).

A total of 66 animals were used for open field testing. Measures included time in the center of the open arena and the number of line crosses, as measures of anxiety-like behavior and locomotion, respectively. There were no significant effects of castration (Fig. 1C, F_1,62_ = 0.1307, *p* = 0.71, *n* = 14–20/group) amongst any of the groups in line crosses, but there was a main effect of vortioxetine treatment (F_1,62_ = 4.528, *p* < 0.05). Vortioxetine increased locomotion overall, and this appeared to be driven primarily by an increase in non-castrated-control rats, although the pairwise comparison of intact controls receiving control diet and those receiving vortioxetine was not significant (*p* = 0.10). There were no significant effects of castration (Fig. 1D, F_1,62_ = 3.389, *p* = 0.07, *n* = 14–20/group) or drug treatment (F_1,62_ = 0.0578, *p* = 0.81) on time in the center of the arena.

### Experiment 2: effects of androgen deprivation and vortioxetine on spatial memory

Fifty-four animals proceeded to the NOL test after habitation, as animals that failed to interact with both objects on the sample phase or at least one object during the test phase were excluded from analysis. Two-way ANOVA revealed significant main effects of castration (Fig. 1E, F_1,50_ = 6.658, *p* < 0.05, *n* = 11–17/group), vortioxetine treatment (*F*_1,50_ = 5.123, *p* < 0.05), and an interaction effect (castration x drug, F_1,50_ = 4.660, *p* < 0.05). Pairwise comparisons using the Holm-Sidak test revealed that surgical castration significantly decreased the discrimination ratio in rats receiving control diet (*p* < 0.05). The deficit in spatial memory was reversed by chronic vortioxetine treatment in castrated animals (*p* < 0.05).

### Experiment 3: effects of androgen deprivation and vortioxetine on afferent-evoked response in the dorsal hippocampus

**Fig. 2:**
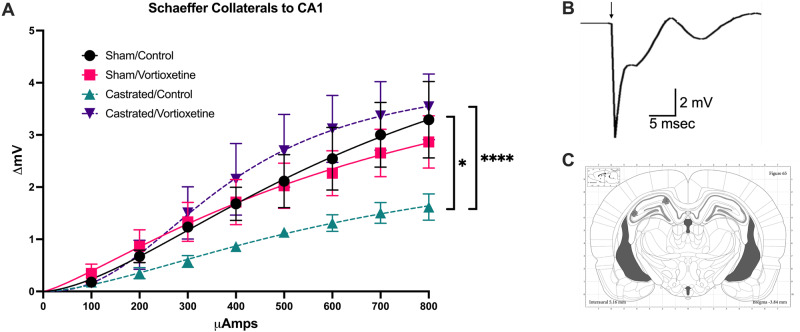
Evoked electrical field potentials recorded in the CA1 region of the dorsal hippocampus in response to stimulation of the afferent from the Schaeffer Collaterals (SC). **A** Pairwise comparison revealed that surgical castration reduced afferent-evoked responsivity compared to sham-control rats (*p < 0.05, *n* = 6/group). Vortioxetine treatment reversed these changes back to baseline levels (***p < 0.0001, castrated-control, *n* = 6 compared to castrated-vortioxetine, *n* = 4). **B** Representative trace of evoked response recorded in CA1 (arrow indicates stimulus artifact). **C** Placement of stimulating and recording electrodes in the Schaeffer Collaterals and CA1 region of dorsal hippocampus, respectively.

For recording in vivo afferent-evoked field potentials in the dorsal hippocampus of anesthetized animals, a total of 22 rats were used. Sum of squares F-test revealed that stimulus-response curves for evoked potentials recorded in the CA1 region in response to stimulation of the Schaeffer Collaterals were significantly altered after castration and vortioxetine treatment (F_(9,164)_ = 6.710, *p* < 0.0001, *n* = 4–6/group, Fig. 2). Pairwise comparisons revealed that castrated rats receiving control diet had an attenuated response in the SC-CA1 pathway in comparison to intact-control rats (F_(7,70)_ = 2.257, *p* < 0.05), and that vortioxetine reversed the attenuated responses in this pathway after castration (F_(7,56)_ = 8.199, *p* < 0.0001).

### Experiment 4: effects of androgen deprivation and vortioxetine on gene expression in the hippocampus

**Fig. 3:**
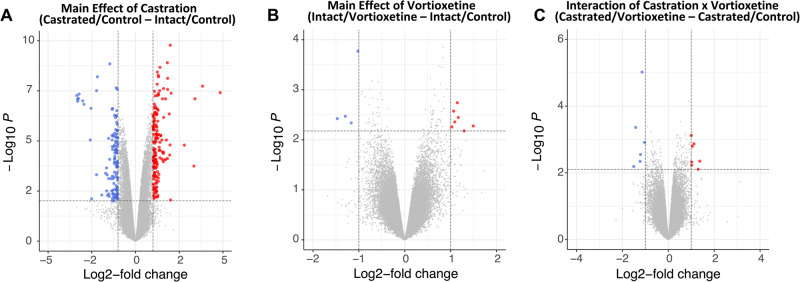
Surgical castration alters gene expression in dorsal Hippocampus (dHipp). Volcano plots depict differential gene expression in dHipp after castration and vortioxetine treatment. Dashed lines represent significance criteria: |FC| ≥ 2 and *p* < 0.01. Genes downregulated are denoted in blue and genes in red were upregulated. **A** In the main effect of castration, there were 279 genes that were differentially affected (165 were upregulated and 114 were downregulated). **B** In the main effect of vortioxetine treatment, 4 genes were downregulated and 7 genes upregulated. **C** Within the interaction of castration x vortioxetine, there were seven genes upregulated and 6 genes downregulated.

**Fig. 4:**
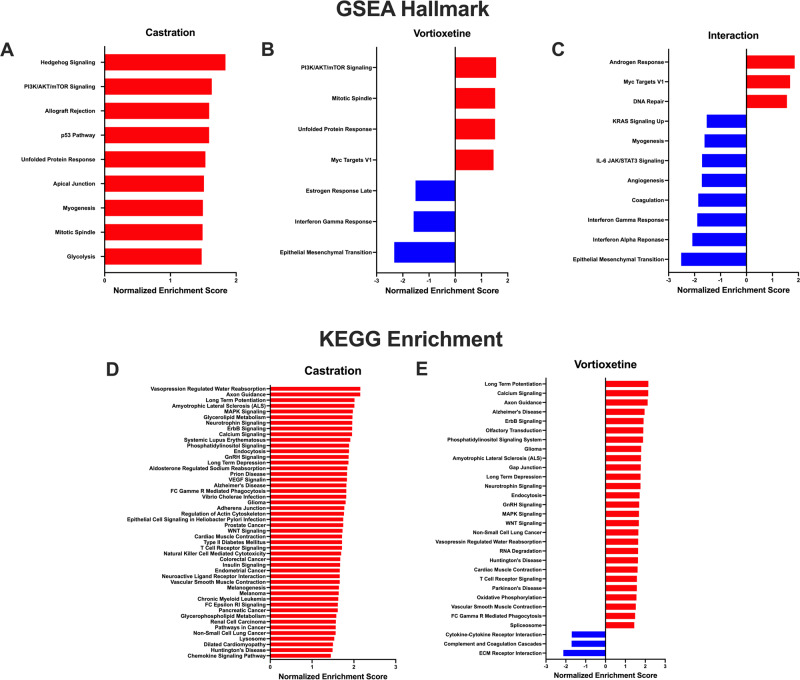
Hallmark and KEGG pathways from Gene Set Enrichment Analysis (GSEA) of genes affected by androgen deprivation and vortioxetine. Pathways were identified by *q* value < 0.05 and ordered by normalized enrichment score. Hallmark pathways were identified in **A** the main effect of castration (castration-control vs. intact-control), **B** the main effect of vortioxetine (intact-vortioxetine vs. intact-control), and **C** the interaction of vortioxetine x castration (castration-vortioxetine vs. castration-control). KEGG pathways were identified in **D** the main effect of castration and **E** the main effect of vortioxetine. Pathways in the interaction did not meet the threshold for significance of *q* < 0.05.

For the microarray analysis, 24 animals were used. Genes were identified based on whether they were differentially expressed based on the background transcriptome. In the dorsal hippocampus, there were 279 genes differentially affected by castration (castrated/control—intact/control, *p* < 0.01, fold change≥ 2 or ≤−2). Of those genes, 165 were upregulated and 114 were downregulated (Fig. 3A). Vortioxetine affected fewer genes. In the main effect of vortioxetine (intact/vortioxetine—intact/control), 11 genes were affected; 4 genes were downregulated, and 7 genes upregulated (Fig. 3B). Similarly, 13 genes total were affected in the interaction of castration x vortioxetine (castrated/vortioxetine—castrated/control); 7 genes were upregulated and 6 were downregulated (Fig. 3C). Differentially expressed genes affected by castration are listed in Table 1, and genes affected by vortioxetine and in the interaction are listed in Table 2. GSEA was then used on the most highly expressed probes (27,440, or ~60% of the total 45,738 probes) rank-ordered by fold change, to determine how differentially expressed genes might be functionally related. Many of these pathways are associated with synaptic plasticity-related processes (Fig. 4A–C, *q* value < 0.05), such as PI3K/Akt/mTOR signaling. Within the KEGG pathways, several pathways were identified in the main effect of castration and vortioxetine treatments, but there were no pathways that reached significance in the interaction (Fig. 4D, E). There were several pathways affected consistently across conditions, including Long-Term Potentiation, Long-Term Depression, Axon Guidance, and MAPK Signaling.

**Table 1:** Effects of androgen deprivation on gene expression in dorsal hippocampus.

Gene name/ID	Log2(FC)	*P* value	Gene name/ID	Log2(FC)	*P* value	Gene name/ID	Log2(FC)	*P* value
Main effect of castration (castrated/control—intact/control)								
DCP_1_0	−3.359	0.000000054	Glrb *	**−1.332**	**0.000019457**	Tvp23b	−1.143	0.000029548
(+)E1A_r60_1	−3.311	0.000000082	**Dsg2**	**−1.321**	**0.007420037**	Kcna2 *	**−1.135**	**0.000010021**
DCP_1_1	−3.305	0.000000104	Ctxn3 *	**−1.320**	**0.000571004**	**Pdcd7**	**−1.130**	**0.000002722**
DCP_1_2	−3.265	0.000000074	44811	−1.312	0.000026796	Rfk *	**−1.130**	**0.000006086**
(+)E1A_r60_n9	−3.226	0.000000046	**Tsc22d3**	**−1.310**	**0.000000244**	DCP_22_0	−1.129	0.000012495
DCP_1_4	−3.021	0.000000100	Ppp1r17 *	**−1.308**	**0.002923115**	Hopx *	**−1.129**	**0.000026010**
(+)E1A_r60_n11	−2.947	0.000000148	Mdfic *	**−1.305**	**0.004472728**	Luc7l3 *	**−1.126**	**0.000026473**
DCP_1_7	−2.590	0.000008919	LOC100912629	−1.288	0.000747769	LOC102550744	−1.119	0.005486318
(+)E1A_r60_a97	−2.544	0.000000237	DCP_20_0	−1.282	0.000005016	A_44_P1360260	−1.109	0.000154546
Tmem72 *	**−2.510**	**0.007744057**	LOC102550328	−1.271	0.004325523	Tspan8 *	**−1.105**	**0.001724714**
**Sulf1**	**−2.229**	**0.000483226**	DCP_20_1	−1.250	0.000005873	Gpr153 *	**−1.102**	**0.000028060**
LOC102552325	−2.227	0.000000031	A_42_P629650	−1.241	0.006149048	Phf10 *	**−1.100**	**0.000000022**
LOC100363289	−2.177	0.000000006	Ppp1cb *	**−1.237**	**0.001322118**	**Ptger3**	**−1.100**	**0.000486050**
**Lilrb3**	**−1.927**	**0.005040186**	A_44_P1152848	−1.235	0.000018732	Zbtb44 *	**−1.096**	**0.000000559**
Sulf1 *	**−1.712**	**0.000410239**	Gpr88	−1.222	0.009585972	Pdgfa *	**−1.090**	**0.000037189**
A_44_P294461	−1.658	0.000223440	**Tmem255a**	**−1.218**	**0.000643838**	**Pdcd7**	**−1.087**	**0.000000549**
Myo5c *	**−1.648**	**0.003145760**	Mybl1 *	**−1.217**	**0.000016766**	LOC100912787	−1.087	0.000031819
Crb3 *	−1.624	0.000490731	Rai14 *	**−1.211**	**0.000130225**	Ly6d *	**−1.086**	**0.002861825**
Olr289	−1.604	0.005245154	Ptgfrn *	**−1.206**	**0.000363580**	Ppat *	**−1.084**	**0.000006743**
A_44_P860459	−1.542	0.000007407	LOC500034	−1.199	0.000110979	A_44_P177106	−1.081	0.000004555
LOC102552576	−1.536	0.000752949	RGD1565959	−1.199	0.000000527	Ets1 *	**−1.077**	**0.000001179**
A_44_P593525	−1.522	0.004426200	LOC316717	−1.192	0.000078774	A_44_P985758	−1.076	0.000113957
Klf5 *	**−1.503**	**0.005835992**	RGD1306148	−1.192	0.000076069	Tcerg1 *	**−1.073**	**0.000202791**
LOC103691368	−1.479	0.000082988	LOC103692770	−1.187	0.002610191	B4galt6	−1.071	0.000018871
A_44_P388174	−1.470	0.000000001	Zfp280d	−1.185	0.000039300	A_44_P176677	−1.071	0.000000307
Fam46a *	−1.399	0.000765256	Tmem196 *	**−1.182**	**0.006645372**	Slco1a2 *	**−1.070**	**0.000040019**
LOC102554942	−1.352	0.003180251	(+)E1A_r60_a20	−1.182	0.000004126	Hif1a *	**−1.065**	**0.000178961**
A_43_P21150	−1.346	0.000000263	Slc7a8 *	**−1.181**	**0.000058397**	Ccdc141 *	**−1.063**	**0.000373571**
Fubp1	−1.345	0.000005384	Fam160a1 *	**−1.174**	**0.004680928**	Slc5a9 *	**−1.063**	**0.006396024**
LOC102553223	−1.337	0.000009035	Tc2n *	**−1.157**	**0.007031053**	Klhl1 *	**−1.062**	**0.002839968**
Baiap2l1 *	**−1.336**	**0.009749783**	A_44_P648481	−1.148	0.000000677	Cgn	−1.057	0.002283754
Trappc2 *	−1.056	0.000660035	Cacng8 *	**1.018**	**0.000075115**	Rbbp4 *	**1.076**	**0.000128378**
Hemgn *	−1.049	0.000196549	Il24	1.020	0.000011484	A_64_P114084	1.077	0.000197679
A_44_P1158527	−1.048	0.003510234	**Anp32a**	**1.021**	**0.000077440**	Atn1	1.080	0.000001001
Cdh16 *	−1.048	0.005332964	A_44_P556745	1.022	0.000011711	A_64_P056703	1.081	0.000002902
Ptprv	−1.046	0.002163285	A_44_P1364899	1.022	0.000002641	Ak5 *	**1.081**	**0.000000033**
**Hif1a**	−**1.044**	**0.000003090**	**Pcbp1**	**1.028**	**0.000002114**	**Gria1**	**1.087**	**0.000373241**
Vom2r46	−1.042	0.004727577	LOC102552929	1.029	0.000185514	Cpa5 *	**1.088**	**0.002460269**
Dhx15 *	**−1.041**	**0.000011149**	A_44_P837263	1.033	0.000551838	Olr604	1.088	0.003650564
A_44_P704874	−1.037	0.000001503	Mgll *	**1.035**	**0.000789768**	A_64_P009178	1.090	0.000000761
A_42_P482051	−1.036	0.000000026	A_44_P330035	1.035	0.000001123	Grin2d *	**1.091**	**0.000054051**
**Cdkn2d**	**−1.035**	**0.000180410**	Arsg *	**1.035**	**0.000001884**	LOC102548371	1.091	0.000096129
A_44_P1155718	−1.031	0.000106181	A_44_P194939	1.036	0.000214844	Gapdh-ps1	1.091	0.000037813
A_44_P134496	−1.030	0.000002990	Mapre3 *	**1.036**	**0.000002619**	A_44_P503167	1.091	0.000487110
Vom1r35	−1.029	0.007863541	Slc8a2	1.037	0.000233388	A_44_P505440	1.092	0.000128723
**Slc8a1**	**−1.026**	**0.000918729**	Extl3 *	**1.037**	**0.000062130**	A_43_P10384	1.094	0.000000387
Mospd1	−1.024	0.004438125	Atp6v0c	1.038	0.000004797	A_43_P10121	1.103	0.000084547
LOC685680	−1.021	0.000126169	Rab3a	1.041	0.000029108	Prkcg *	**1.108**	**0.000386664**
A_43_P19304	−1.017	0.000007029	Csrp1 *	**1.045**	**0.002809659**	Galnt10 *	**1.109**	**0.002419290**
(+)E1A_r60_a22	−1.011	0.000015539	Limd2 *	**1.046**	**0.000541715**	LOC103694359	1.111	0.000211324
A_44_P261663	−1.008	0.000713982	Unc5a	1.047	0.000024450	LOC102555479	1.111	0.000012816
Tmem196	−1.003	0.007893654	Syngr1	1.059	0.000000505	A_44_P1153530	1.116	0.000619762
A_44_P239345	1.001	0.001658717	Hmx1 *	**1.060**	**0.000030047**	Gnas *	**1.121**	**0.000025843**
Kif1b *	**1.002**	**0.000001844**	LOC102554855	1.062	0.007443989	**Cfap43**	**1.123**	**0.000000186**
A_44_P122429	1.006	0.000002587	**Ptprn2**	**1.064**	**0.000000228**	Nacc1 *	**1.125**	**0.000000057**
A_44_P1159153	1.007	0.000000539	Glra2 *	**1.068**	**0.000001483**	A_44_P1156801	1.125	0.000010486
Zfp958	1.009	0.001508251	A_44_P944361	1.068	0.000001016	Cers1	1.133	0.000000376
Foxo1 *	**1.010**	**0.000000660**	A_44_P1363214	1.069	0.004653488	A_44_P1367985	1.135	0.000544312
**Cntfr**	**1.012**	**0.005736210**	**Fibcd1**	**1.071**	**0.000559195**	A_64_P101983	1.137	0.000054496
Pde3a *	1.014	0.002322651	Dlx3	1.075	0.000054847	Camk2a *	**1.139**	**0.000129963**
A_44_P237564	1.016	0.000099113	LOC102554145	1.076	0.006526812	Bcl11b *	**1.142**	**0.001301627**
A_44_P1364435	1.017	0.000098959	A_64_P147586	1.076	0.000000145	Crebbp *	**1.144**	**0.000088531**
Atp1a3 *	**1.146**	**0.000007877**	Cym	1.222	0.000004822	A_44_P1152546	1.406	0.000029247
A_44_P1364694	1.147	0.000050872	Csrnp2 *	**1.228**	**0.000623765**	A_64_P017723	1.438	0.000005293
S1pr5 *	1.148	0.002539634	LOC102555044	1.233	0.000000432	Aes	1.440	0.000005510
Clmp *	**1.149**	**0.001954947**	LOC103691813	1.236	0.000007215	Zc2hc1b	1.503	0.000080619
A_44_P1361950	1.150	0.000000231	**Slc24a5**	**1.237**	**0.000000053**	Ccl22 *	**1.509**	**0.000008980**
Gng3	1.151	0.000038855	A_44_P1041449	1.237	0.000000015	LOC100911409	1.536	0.000035945
LOC100912383	1.158	0.000231119	**Nfix**	**1.239**	**0.000003119**	A_44_P355294	1.546	0.000000079
LOC102555855	1.160	0.000575882	Cfl1 *	**1.249**	**0.000000004**	Hes7 *	**1.586**	**0.000108971**
A_44_P221213	1.162	0.000000231	A_44_P912004	1.249	0.000147097	A_44_P1363943	1.590	0.000041950
Gria1 *	**1.163**	**0.000006308**	Atp6v0c	1.252	0.000000255	LOC102553671	1.597	0.000036389
Lrrn4 *	**1.163**	**0.000000421**	Gngt1	1.257	0.006322879	LOC103691795	1.604	0.000000668
LOC103690307	1.166	0.000098665	Znrf4 *	**1.260**	**0.000344007**	A_44_P462228	1.604	0.000000024
Rassf2 *	**1.167**	**0.000010655**	A_64_P158343	1.263	0.000005071	LOC103690864	1.635	0.000089378
LOC102549602	1.170	0.000082615	Ube2g2	1.272	0.000007383	A_44_P169952	1.719	0.000000079
Ube2d1 *	**1.171**	**0.000001592**	Nsmf *	**1.278**	**0.000001033**	Cnih2	1.729	0.000083115
A_44_P201693	1.176	0.000004500	Shisa4 *	**1.282**	**0.005315913**	A_44_P363937	1.764	0.000008941
Nptxr	1.177	0.000126721	A_44_P122061	1.293	0.000000294	LOC103692432	1.820	0.000000001
Atp2a1 *	**1.179**	**0.003887588**	A_64_P123155	1.301	0.000000005	Zdhhc18	1.826	0.000075049
LOC102553558	1.180	0.000007378	A_44_P527614	1.305	0.000704143	LOC103695206	1.835	0.000000007
Capn15 *	**1.184**	**0.000932570**	Scn1b *	**1.307**	**0.001017811**	LOC102551957	1.838	0.000000027
Nek8	1.188	0.000001862	Eno1 *	**1.310**	**0.000000043**	Rn45s	1.932	0.000000449
LOC102547434	1.195	0.000004377	Srpr	1.317	0.000000051	LOC102723236	1.957	0.000050680
**Cotl1**	**1.199**	**0.003224862**	**Zfhx2**	**1.317**	**0.000000006**	LOC103693243	1.974	0.000015859
RGD1561017	1.201	0.006186444	Phyhip *	**1.327**	**0.000004801**	Fam20a *	**1.982**	**0.000000000**
Thy1	1.207	0.000000501	A_44_P574530	1.338	0.000463834	Tph1 *	**2.004**	**0.008882666**
LOC103692246	1.210	0.003204180	Prss35 *	**1.350**	**0.000030099**	LOC682526	2.037	0.000000041
Neurod2 *	**1.213**	**0.000189934**	A_64_P125130	1.355	0.000020770	Rn5–8s	2.793	0.000016169
S100a7a	1.214	0.001279380	A_44_P413787	1.381	0.000000031	LOC310926	3.340	0.000179384
44809	1.218	0.000002062	Olfm1 *	**1.394**	**0.000011652**	Rn45s	3.402	0.000000079
LOC103690992	1.220	0.002043341	A_44_P1155460	1.394	0.000069586	Rn18s	3.834	0.000000018
LOC680959	1.222	0.000114768	A_64_P088257	1.399	0.000000002	Rn45s	4.844	0.000000038

In instances where gene name was not available, gene ID was provided. Asterisk indicates genes containing an androgen response element (ARE). Bold indicates genes known from the literature to be androgen responsive.

**Table 2:** Effects of vortioxetine on gene expression in dorsal hippocampus.

Gene name/ID	Log2(FC)	*P* value
A: Main effect of vortioxetine (intact/vortioxetine—intact/control)		
LOC103694566	−1.021	0.000168945
LOC102546359	1.145	0.001821412
LOC102548769	1.066	0.002670715
A_44_P1363336	−1.294	0.003389937
Neurod4	1.167	0.003581896
A_44_P415021	−1.470	0.003774881
Cst5 *	**1.090**	**0.004395340**
LOC103693058	−1.168	0.004599649
A_44_P241722	1.493	0.005289019
S100a7a	1.027	0.005523844
LOC102557151	1.295	0.006663463

B: Interaction of castration x vortioxetine (castrated/vortioxetine—castrated/control)		
LOC102547056	−1.143	0.000009518
LOC103692843	−1.422	0.000434028
Sftpb *	**1.000**	**0.000757574**
A_44_P217744	−1.039	0.001220077
**Hoxb3**	**1.117**	**0.001350312**
Olr1236	1.049	0.001591478
Clul1 *	**−1.209**	**0.002828433**
A_44_P374593	1.373	0.004464380
LOC102554944	−1.229	0.004532593
A_44_P154016	1.020	0.004698516
LOC102550135	1.016	0.005920719
Olr584	−1.507	0.006452363
A_44_P1368178	1.302	0.007802496

In instances where gene name was not available, gene ID was provided. Asterisk indicates genes containing an androgen response element (ARE). Bold indicates genes known from the literature to be androgen responsive.

## Discussion

Quality of life is an essential consideration in the care of cancer patients. ADT produces significant impairments in cognitive domains associated with hippocampal function, specifically in spatial learning and memory, and these deficits can profoundly impact patients’ quality of life. As prostate cancer is most common in older men, these cognitive deficits can exacerbate other factors that may contribute to neurocognitive decline. Cognitive impairment not only impacts quality of life for cancer survivors, but can also affect their caregivers and families [29]. In prostate cancer patients, ADT increases the risk of Alzheimer’s disease and dementia [30, 31]. Therefore, finding ways to mitigate the detrimental effects of ADT on the brain may offer important advances to improve patients’ quality of life. Unfortunately, the brain mechanisms impacted by ADT, and the mechanisms by which cognition is disrupted have not been established. Furthermore, treatments for patients experiencing cognitive decline are largely ineffective. The purpose of this study was to investigate the cognitive decline associated with ADT, to understand the mechanisms that underlie the impairment, and to test a potentially novel therapeutic candidate.

To investigate the effects of ADT on cognition mediated by the hippocampus, we used the NOL test to assess visuospatial memory, as this test is specifically mediated by the dorsal hippocampus, and is largely unaffected by lesions in the prefrontal cortex [22]. Previous studies have shown that surgical castration induced deficits in spatial memory tasks such as the radial arm maze [32, 33], but chemical castration with bicalutamide and goserelin produced mixed results [32]. Likewise, not all types of spatial learning tasks are affected by castration [33]. This could be due to the involvement of brain regions other than the hippocampus in many of these tests, leading to inconsistent results. Therefore, we chose to use the NOL test, as this variation of the novel object paradigm is known to be specifically dependent on the hippocampus [22] and is therefore less subject to confounding effects by activity in other brain regions, such as the medial prefrontal cortex (mPFC), which we have shown previously to be dysfunctional after ADT [14]. In the current study, ADT impaired visuospatial memory on the NOL test, and vortioxetine reversed the deficit in castrated rats, restoring performance comparable to intact controls. This suggests that vortioxetine may be a potential therapeutic candidate to treat hippocampal-related cognitive decline associated with ADT in prostate cancer patients.

While the human literature on ADT-induced cognitive impairment is sparse, animal studies have provided insight to the effects of androgen depletion on brain processes. Androgens are known to broadly affect brain activity and cognition [34]. The AR is widely expressed in the brain, and regions such as the prefrontal cortex, thalamus, and hippocampus contain some of the highest densities [34]. Of note, the CA1 region of the hippocampus exhibits particularly high density of the AR, and this region is known to underlie visuospatial cognition [22, 35]. Castration with either surgical gonadectomy or administration of the AR antagonist, flutamide, in rats significantly reduced AR mRNA expression within this same region [35]. The hippocampus is therefore a crucial site for investigation of ADT-induced cognitive deficits. To further understand the mechanism by which ADT and vortioxetine treatment affected behavior, we investigated functional changes in responsivity to afferent input as a readout of synaptic efficacy within the dorsal hippocampus. Previously, we have shown that surgical castration disrupted the response of the mPFC to ventral hippocampal input but did not affect the afferent-evoked response to medial dorsal thalamic input to the mPFC [14]. Here, we found that castration attenuated responses evoked in the CA1 region of dorsal hippocampus by stimulation of the Schaeffer Collateral afferent, and these changes were reversed by chronic vortioxetine treatment. Together with our previous result, it is thus possible that the detrimental effects of ADT may originate primarily by disruption of local circuit function in the hippocampus. In the dorsal hippocampus, this may directly impair visuospatial cognition, whereas alterations in ventral hippocampal function may further disrupt executive processes modulated by its projection to the mPFC.

The androgen receptor is an important transcription factor in modulating plasticity in the brain [15–17, 36, 37]. Therefore, we assessed whole genome expression profiles using a microarray to identify possible changes at the transcriptome level. Castration had a significant effect on gene expression in the dorsal hippocampus. Of note, many of the affected genes contain androgen response elements, consistent with a direct effect on transcription that would be expected with depletion of testosterone. Genes with known androgen regulation, which were identified through the literature [38–41] are bolded in Tables 1 and 2. However, many of the affected genes did not contain androgen response elements, suggesting that they are likely downstream of directly androgen responsive genes. Several affected genes, such as Gabbr1, Got1, Kcnj4, and Rnd1, are highly expressed in the cortex and hippocampus and have been linked to regulation of neuronal function and cognition [42–45]. Genes such as Camk2a, Crebbp, and Grik4, which were also significantly affected, are important for neurotransmission and may be disrupted in neuropsychiatric conditions [46–48]. Therefore, the detrimental effects of ADT on cognition in the hippocampus may be attributable to changes in gene expression that disrupt neuronal plasticity necessary for optimal cognitive functioning. Further examination of these genes using gene set enrichment analysis revealed that gene expression changes were associated with signaling pathways that are known to participate in cognitive processes, such as PI3K/Akt/mTOR and JAK/STAT3 signaling. Changes consistent with this were seen in the KEGG pathways, as many synaptic plasticity-related pathways were identified in the castration and vortioxetine conditions. These signaling cascades affected by both vortioxetine and castration provide potential targets for future investigation.

The multimodal activity of vortioxetine provides this drug with distinctive qualities in comparison to other antidepressants and may underlie its unique capacity to enhance cognition. For example, vortioxetine is an antagonist at the 5-HT3 receptor, with high occupancy at therapeutic doses. Studies have shown that ondansetron, also a 5-HT3 antagonist, can enhance the antidepressant activity of SSRIs, improves cognition, and has anxiolytic effects on its own [49–52]. Certainly, the modulation of serotonin transmission is critically important to antidepressant action [53]. But antidepressants have also been shown to interact directly with the TrkB receptor [54], and the endogenous ligand for this receptor, brain derived neurotrophic factor (BDNF) is also important for cognition [55]. It is worth noting that in the KEGG analysis, the phosphatidylinositol signaling system was affected by both castration and vortioxetine treatment. However, in the context of cancer, it may be undesirable to target BDNF and TrkB directly by systemic treatment, as both are expressed in prostate tumors and their dysregulation can contribute to tumor progression [56, 57]. Furthermore, the Val66Met BDNF gene polymorphism is associated with neurocognitive alterations, particularly in executive function [57]. In clinical studies, Met/Met carriers seem to be protected from chemotherapy-induced cognitive decline [58] whereas Val/Met carriers were susceptible to impairments [59]. Thus, modulation of BDNF and TrkB signaling are important factors in cancer and cancer-therapy and may also be important to cognitive decline associated with cancer treatment. The multimodal pharmacological activity of vortioxetine targeting neurotransmitter receptors may selectively modulate, for instance, BDNF-related signaling pathways in the brain, including PI3 kinase-Akt-mTOR signaling, without promoting cancer peripherally, or interfering with the anti-cancer efficacy of ADT.

In contrast to a previous report that vortioxetine regulated gene expression in naïve animals [60], there were relatively few significant changes in gene expression in castrated animals after vortioxetine treatment. This discrepancy could be due to a few reasons. The study that showed vortioxetine regulated gene expression involved drug treatment for one month, and multiple doses were used, but not all doses produced similar effects on gene expression. Also, that study used a larger sample size, which provided more power to the analysis. Furthermore, it is important to note that changes in gene expression may occur transiently, at early stages of chronic antidepressant treatment. This has been reported in studies of antidepressant effects on serotonin transporter (SERT) mRNA expression using fluoxetine [61] and p-chlorophenylalanine [62]. Similarly, transient changes in gene expression may occur with vortioxetine treatment. It must also be recognized that either castration or vortioxetine could affect processes, including gene expression, in other brain regions that secondarily alter function of the hippocampus. For example, we have previously shown that cognitive flexibility mediated in the medial prefrontal cortex (mPFC) was impaired after surgical castration, with corresponding changes in gene expression in the mPFC [14]. The mPFC does not project directly to the dorsal hippocampus, but it could influence hippocampal function via, for example, a frontocortical-thalamic-hippocampal projection. Other brain regions that provide input to the hippocampus could also contribute to effects observed after castration or systemic vortioxetine treatment. Of course, the multiplicity of brain regions potentially affected by vortioxetine may be an advantage for therapeutic efficacy, as plasticity induced in many brain regions may contribute to beneficial effects on many behaviors impaired by androgen deprivation.

Nonetheless, the general lack of effects of vortioxetine on gene expression suggests that rather than reversing the detrimental effects of ADT on gene expression to rescue cognitive deficits, vortioxetine may instead initiate signaling processes in the brain that compensate for these changes. For example, chronic vortioxetine has been shown to increase phosphorylation of the GluA1 subunit of the AMPA receptor and of actin depolymerizing factor (ADF)/cofilin, without altering total protein levels [63]. Vortioxetine enhances long-term potentiation (LTP) in the hippocampus [63], which is dependent on synaptic plasticity and maturation of synaptic spines. The processes that underlie LTP require activity-dependent changes in plasticity-related signaling factors, such as the PI3K signal transduction pathway. Our enrichment analysis revealed pathways affected by both ADT and vortioxetine, including PI3K/MAPK/mTOR signaling, mitotic spindle, and unfolded protein response, which were upregulated. KEGG analysis also identified relevant pathways affected by both manipulations, e.g., long term potentiation and axon guidance. Given that PI3K and related signaling processes involved in plasticity were affected by both castration and vortioxetine, it may be a potential candidate for future investigation into mechanisms of ADT-induced cognitive impairment and effective therapeutic intervention.

There are some limitations to these initial experiments. One is that the animals in this study were healthy and did not have cancer. Cancer itself activates inflammatory and metabolic processes that can affect cognition [64, 65]. It can also produce symptoms of fatigue [66], which may exacerbate or contribute to cognitive impairment. The purpose in using cancer-free rats in these initial studies was to investigate mechanisms underlying the cognitive impairments induced by ADT without the potentially confounding effects of cancer pathophysiology. In our previous study, we showed that vortioxetine did not directly promote the growth of either androgen-dependent or -independent prostate cancer cells in vitro, and did not interfere with the anti-proliferative activity of the androgen-receptor antagonist enzalutamide [14]. Thus, future studies will test the potential influence of cancer on the cognitive impairments associated with ADT and the beneficial effects of vortioxetine using a syngeneic rat prostate cancer model. Secondly, we used surgical castration to produce testosterone depletion, which is less translationally relevant than other methods of chemical castration. However, this allowed us to isolate the effects of testosterone depletion alone on cognition, without the potentially confounding effects of chemical castration using a pharmacological intervention that may also have off-target effects. In ongoing experiments, we are replicating the effects of surgical castration using chemical castration with the GnRH antagonist degarelix, which is more translationally relevant.

In summary, the results of these experiments indicate that ADT alters synaptic efficacy in the hippocampus and compromises visuospatial cognition, providing a valid model of cognitive impairment after ADT that can be used to test the utility of potential therapeutic interventions to restore cognitive function and improve quality of life for cancer survivors. Further, our results suggest that vortioxetine, a novel FDA-approved multimodal antidepressant, may be one such candidate. Vortioxetine reversed deficits in hippocampal response and visuospatial cognition induced by ADT. Thus, vortioxetine, a relatively safe and well-tolerated drug, may have potential utility for treating cognitive impairment associated with ADT in prostate cancer survivors.
